# Supplementary material for: Sphingosine-1-Phosphate Levels Are Higher in Male Patients with Non-Classic Fabry Disease
Source: J Clin Med. 2022 Feb 24;11(5):1233. doi: 10.3390/jcm11051233 (PMC8911241; doi:10.3390/jcm11051233)
Supplement: Supplementary file 1 [file jcm-11-01233-s001.zip › jcm-1583311-supplementary.pdf]

Supplementary Table S1. Characteristics of patients.

Patient*	Age (years)	*GLA* variant (cDNA)	*GLA* variant (Protein)	FFABRY phenotype	Cerebral Stroke	Acroparesthesia (past or present)	creatinine (µM)	eGFR	HCM	IVS Thickness	Treatment exposure**	Cumulative exposure (years)	Ab status	lysoGb3 (ng/ml)	S1P (ng/ml)	FFABRY total [1]	MSSI total [2]
P1	17,1	c.802-3_802-2del	p.?	NonClassic	noStroke	Pain	65	136,177	noHCM	11	0	0	Ab-	73	268,12	4	7
P2	17,5	c.902G>A	p.Arg301Gln	Classic	noStroke	Pain	64	136,637	noHCM	9	B	0,42	Ab+		210,20	1	14
P3	20,3	c.486C>T	p.Trp162Cys	Classic	noStroke	Pain	80	122,267	noHCM	no recent data	0	0	Ab-		92,25	0	2
P4	21,5	c.137A>G	p.His46Arg	Classic	noStroke	Pain	58	138,355	noHCM	11	A	3,62	Ab-	10,6	152,01	3	14
P5	24,9	c.902G>A	p.Arg301Gln	NonClassic	noStroke	noPain	75	121,590	noHCM	no recent data	0	0	Ab-	2	219,18	2	4
P6	27,7	c.901C>T	p.Arg301*	Classic	noStroke	Pain	60	130,632	noHCM	no recent data	AB	5,76	Ab+	48,8	158,50	1	19
P7	27,9	c.729G>C	p.Leu243Phe	Classic	Stroke	Pain	66	125,452	noHCM	no recent data	AB	12,61	Ab+		296,80	2	18
P8	30,1	c.1246C>T	p.Gln416*	Classic	noStroke	Pain	71	119,893	noHCM	no recent data	B	15,50	Ab-	10,2	63,05	1	17
P9	31,9	c.875C>T	p.Ala292Val	Classic	noStroke	Pain	67	121,241	noHCM	10	A	11,33	Ab+	15	170,10	1	8
P10	32,7	c.884T>G	p.Phe295Cys	Classic	noStroke	Pain	renal transplant	renal transplant	noHCM	11	BMIG	3,89	Ab+	10	107,80	6	29
P11	33,2	c.548G>C	p.Gly183Ala	Classic	noStroke	Pain	80	111,689	noHCM	9,4	AB	13,14	Ab+	76	22,16	1	11
P12	33,4	c.847C>T	p.Gln283*	NonClassic	noStroke	Pain	72	116,464	HCM	14	B	13,31	Ab+	48	199,11	1	14
P13	34,0	c.424T>C	p.Cys142Arg	Classic	noStroke	Pain	83	106,186	HCM	14	A	11,90	Ab-	33,3	68,61	1	12
P14	34,7	c.644A>G	p.Asn215Ser	NonClassic	noStroke	Pain	68	118,153	HCM	22	B	1,32	Ab-	2,7	290,34	1	20
P15	35,0	c.337T>C	p.Phe113Leu	NonClassic	noStroke	noPain	63	121,656	noHCM	no recent data	0	0	Ab-	3	191,44	0	0
P16	41,1	c.1069-1079del	p.Gln357Trpfs*14	Classic	noStroke	Pain	89	92,867	noHCM	no recent data	AB	12,22	Ab+	18,9	197,06	1	24
P17	43,0	c.692A>G	p.Asp231Gly	NonClassic	Stroke	Pain	73	108,267	HCM	17	B	0,04	Ab-	16,7	200,28	4	26
P18	43,3	c.169C>T	p.Gln57*	Classic	no data	Pain	renal transplant	renal transplant	HCM	17	B	13,83	Ab+	31,3	270,59		29
P19	43,8	c.233C>G	p.Ser78*	Classic	noStroke	Pain	renal transplant	renal transplant	HCM	24	B	15,16	Ab+	41	221,39	8	45
P20	44,4	no data	no data	Classic	noStroke	Pain	64	113,163	noHCM	7,4	AB	14,86	Ab-	10,1	144,34	4	7
P21	45,3	c.658C>T	p.Arg220*	NonClassic	noStroke	Pain	82	99,526	HCM	no recent data	AB	15,35	Ab-	7,1	189,61	1	14
P22	46,1	c.901C>T	p.Arg301*	Classic	noStroke	Pain	77	103,600	noHCM	11	A	5,69	Ab+	60	193,17	3	27
P23	46,3	c.680G>A	p.Arg227Gln	Classic	noStroke	Pain	109	70,077	HCM***	8,7	AB	11,44	Ab-	18,4	168,62	6	34
P24	46,3	c.1010T>C	p.Phe337Ser	Classic	noStroke	Pain	96	81,700	HCM	no recent data	B	4,31	Ab-	3,3	200,98	5	20
P25	47,3	c.847C>T	p.Gln283*	NonClassic	noStroke	noPain	77	102,749	HCM	14	B	13,05	Ab+	25,4	189,64	2	12
P26	47,6	c.644A>G	p.Asn215Ser	NonClassic	noStroke	Pain	59	114,361	HCM	no recent data	AB	2,21	Ab-		186,98	4	28
P27	47,7	c.522T>A	p.Cys174*	NonClassic	noStroke	Pain	120	61,789	HCM	no recent	B	0,27	Ab+	21,7	202,88	3	12

										data							
P28	48,1	c.334C>T	p.Arg112Cys	NonClassic	noStroke	Pain	71	105,607	HCM	no recent data	AB	13,42	Ab-	24,6	219,44	3	21
P29	49,2	c.802-3_802-2del	p. ?	Classic	noStroke	Pain	240	26,435	HCM	24	0	0	Ab-	109,3	125,55	8	35
P30	49,8	c.847C>T	p.Gln283*	Classic	noStroke	Pain	renal transplant	renal transplant	HCM	no recent data	B	11,15	Ab+	84,2	234,47	6	31
P31	51,8	c.644A>G	p.Asn215Ser	NonClassic	noStroke	noPain	93	81,708	HCM	17	A	0,07	Ab-		178,13	4	22
P32	57,6	c.713G>A	p.Ser238Asn	NonClassic	noStroke	noPain	81	92,648	HCM	23	B	6,59	Ab-	2,4	217,33	2	16
P33	58,1	c.644A>G	p.Asn215Ser	NonClassic	noStroke	Pain	62	104,106	HCM	22	0	0	Ab-	8,5	227,93	4	26
P34	59,1	c.758T>C	p.Ile253Thr	NonClassic	noStroke	noPain	126	53,737	HCM	13	A	0,65	Ab+	10,8	197,31	7	23
P35	59,4	c.334C>T	p.Arg112Cys	Classic	noStroke	Pain	252	23,209	HCM	no recent data	B	5,57	Ab-	12,5	190,70	8	37
P36	59,8	c.1087C>T	p.Arg363Cys	NonClassic	noStroke	noPain	106	65,944	HCM	no recent data	A	4,24	Ab-	0,8	114,87	6	15
P37	60,8	c.713G>A	p.Ser238Asn	NonClassic	noStroke	noPain	81	90,640	HCM	34	B	7,46	Ab-	2,2	192,46	6	26
P38	60,9	c.1010T>C	p.Phe337Ser	NonClassic	noStroke	Pain	118	57,446	HCM	no recent data	B	4,84	Ab-	5,8	229,54	6	20
P39	61,4	c.1087C>T	p.Arg363Cys	NonClassic	noStroke	noPain	115	59,079	HCM	19	A	4,40	Ab-	1,9	186,95	6	24
P40	67,6	c.486G>C	p.Trp162Cys	NonClassic	noStroke	noPain	97	69,482	HCM	no recent data	B	7,17	Ab-	22,5	230,23	5	1
P41	74,2	c.644A>G	p.Asn215Ser	NonClassic	noStroke	noPain	201#	27,493	HCM	27	B	1,65	Ab-	6,7	273,19	6	34

Ab +/- : antibody positive/negative; eGFR : (CKD EPI) ml/min/1,73m²; HCM Hypertrophic cardiomyopathy; IVS interventricular septum

* Patient identification numbers are different from the figure 3

**Treatment exposure: A: agalsidase alfa, B: agalsidase beta; Mig: migalastat

***HCM was assessed with cardiac echography and cardiac MRI with hypertrophy in the lateral and postero lateral wall

elevated creatinine in the context of cardiac failure, hypertension and hepatitis B virus related cryoglobulinemic vasculitis.

References

1. Mauhin, W.; Benveniste, O.; Amelin, D.; Montagner, C.; Lamari, F.; Caillaud, C.; Douillard, C.; Dussol, B.; Leguy-Seguin, V.; D'Halluin, P.; et al. Cornea Verticillata and Acroparesthesia Efficiently Discriminate Clusters of Severity in Fabry Disease. *PLoS ONE* **2020**, *15*, e0233460, doi:10.1371/journal.pone.0233460.
2. Whybra, C.; Kampmann, C.; Krummenauer, F.; Ries, M.; Mengel, E.; Miebach, E.; Baehner, F.; Kim, K.; Bajbouj, M.; Schwarting, A.; et al. The Mainz Severity Score Index: A New Instrument for Quantifying the Anderson-Fabry Disease Phenotype, and the Response of Patients to Enzyme Replacement Therapy. *Clin. Genet.* **2004**, *65*, 299–307, doi:10.1111/j.1399-0004.2004.00219.x.
